# Defining “mutation” and “polymorphism” in the era of personal genomics

**DOI:** 10.1186/s12920-015-0115-z

**Published:** 2015-07-15

**Authors:** Roshan Karki, Deep Pandya, Robert C. Elston, Cristiano Ferlini

**Affiliations:** Danbury Hospital Research Institute, Western Connecticut Health Network, 131 West Street, Danbury, CT 06810 USA; Department of Epidemiology and Biostatistics, Case Western Reserve University School of Medicine, Cleveland, OH USA

**Keywords:** Personal genomics, Precision medicine, DNA sequencing, DNA variants, Human genome

## Abstract

**Background:**

The growing advances in DNA sequencing tools have made analyzing the human genome cheaper and faster. While such analyses are intended to identify complex variants, related to disease susceptibility and efficacy of drug responses, they have blurred the definitions of mutation and polymorphism.

**Discussion:**

In the era of personal genomics, it is critical to establish clear guidelines regarding the use of a reference genome. Nowadays DNA variants are called as differences in comparison to a reference. In a sequencing project Single Nucleotide Polymorphisms (SNPs) and DNA mutations are defined as DNA variants detectable in >1 % or <1 % of the population, respectively. The alternative use of the two terms mutation or polymorphism for the same event (a difference as compared with a reference) can lead to problems of classification. These problems can impact the accuracy of the interpretation and the functional relationship between a disease state and a genomic sequence.

**Summary:**

We propose to solve this nomenclature dilemma by defining mutations as DNA variants obtained in a paired sequencing project including the germline DNA of the same individual as a reference. Moreover, the term mutation should be accompanied by a qualifying prefix indicating whether the mutation occurs only in somatic cells (somatic mutation) or also in the germline (germline mutation). We believe this distinction in definition will help avoid confusion among researchers and support the practice of sequencing the germline and somatic tissues in parallel to classify the DNA variants thus defined as mutations.

## Background

The human genome consists of over 3 billion base pairs which reside in every nucleated cell of the body [1, 2]. The genome, which has remained well conserved throughout evolution, is at least 99.5 % identical between any two humans on the planet [3]. Modern genomic tools have revealed that it is more complex, diverse, and dynamic than previously thought, even though the genetic variation is limited to between 0.1 % [4–6] and 0.4 % [7] of the genome. Sequence variations, even in non-protein coding regions of the DNA, have begun to alter our understanding of the human genome. While some studies have linked certain variants to being predictive of disease susceptibility and drug response, the majority of diseases have a very complex genetic signature (reviewed in [8, 9]). Biomedical research is shifting towards understanding the functional importance of many such variations and their association with human diseases.

At the heart of these novel discoveries are the modern DNA sequencing tools, which continue to evolve at a rapid pace. The new sequencing technologies continue to become cheaper and more precise, and facilitate novel medical and biological breakthroughs all over the world [10, 11]. Scientific research has become nearly inconceivable without employing sequencing technology but, with the progress of technology and the increasing sequencing of individuals, a massive amount of data is being generated. However, any data without context and analysis is useless. The data from sequencing must be carefully annotated, securely stored, and easily accessible from repositories when needed. Such arduous tasks require functional collaboration among clinicians, researchers, and health professionals [12].

In a recent thread in the ResearchGate portal [13], an ongoing discussion on the difference between a mutation and a polymorphism elicited a response from more than three hundred participants from various scientific backgrounds. The variety of responses prompted us to write this document as a paper aimed at stimulating the discussion further and possibly finding a consensus on the usage of the terms mutation and polymorphism in the context of a reference sequence in a personal genome project.

## Discussion

### The rise of genomics and its impact on human health

Established in 1990, the Human Genome Project was one of the most expensive and collaborative ventures ever undertaken in science. Ten years since its completion, it has continued to provide a wealth of novel information, the implications of which are not yet fully understood [8]. The open-access nature of the project has stimulated scientists, as well as scientific companies, to develop better sequencing tools and accompanying analytical software. The ensuing innovations have helped to mark down the price of whole genome sequencing over the years, from nearly $3 billion at its inception to under $3,000, making it accessible to researchers from different biomedical disciplines [14].

Sequencing tools will play an important role in the development of personalized medicine. Some sequencing technologies are already used in clinics to test genetic conditions, diagnose complex diseases, or screen patient samples for rare variants. These tests allow health professionals to accurately diagnose a disease and prescribe appropriate medication specific to the patient [15, 16]. With the recent support of NIH grants in the US, neonatal sequencing is being explored to probe rare and complex disorders of newborn babies [17, 18]. There are technologies in development that allow non-invasive ways of sequencing a genome of an unborn child [19]. Personalized genome sequencing will transform the future of the healthcare landscape. However, the rise in the number of sequenced genomes is creating new problems. In particular, the way the genome analysis software works is through comparison of the obtained sequences with a reference. Because the human genome is different between different individuals, what is the reference sequence? What is the threshold to distinguish common from rare DNA variants?

Amid all these interesting implications of genome sequencing, the debate concerning the correct use of scientific terminology remains. Specifically, the nomenclature “mutation” and “polymorphism”, and also “point mutation” versus “SNP”, can be independently used to describe the same event, namely a difference in the sequence as compared with a reference. From a strictly grammatical and etymological point of view, a mutation is an event (of mutating) and a polymorphism is a condition or quality (of being polymorphic); but these terms by extension quickly came to mean the resulting event or condition itself. In principle, a point DNA variant can be labeled as a mutation or SNP. Since no clear rules are available, currently used software tools used for genome sequencing make no assignment and label the difference simply as DNA variant, blurring the distinction between the two categories.

### “Mutation” and “polymorphism”: earlier definitions

The uniform and unequivocal description of sequence variants in human DNA and protein sequences (mutations, polymorphisms) were initiated by two papers published in 1993 [20, 21]. In this context, any rare change in the nucleotide sequence, usually but not always with a disease causing attribute, is termed a “mutation” [22]. This change in the nucleotide sequence may or may not cause phenotypic changes. Mutations can be inherited from parents (germline mutations) or acquired over the life of an individual (somatic mutations), the latter being the principal driver of human diseases like cancer. Germline mutations occur in the gametes. Since the offspring is initially derived from the fusion of an egg and a sperm, germline mutations of parents may also be found in each nucleated cell of their progeny. Mutations usually arise from unrepaired DNA damage, replication errors, or mobile genetic elements. There are several major classes of DNA mutations. A point mutation occurs when a single nucleotide is added, deleted or substituted. Along with point mutations, the whole structure of a chromosome can be altered, with chromosomal regions being flipped, deleted, duplicated, or translocated [23]. Another kind of DNA mutation is defined as “copy number variation”. In this case, the expression of a gene is amplified (or reduced) through increased (decreased) copy number of a locus allele [24, 25].

A variation in the DNA sequence that occurs in a population with a frequency of 1 % or higher is termed a polymorphism [26]. The higher incidence in the population suggests that a polymorphism is naturally occurring, with either a neutral or beneficial effect. Polymorphisms can also be of one or more nucleotide changes, just like mutations. The SNP exemplifies the commonest polymorphism, thought to arise every 1,000 base pairs in the human genome, and is usually found in areas flanking protein-coding genes [27] – regions now recognized as critical for microRNA binding and regulation of gene/protein expression [28]. However, SNPs can also occur in coding sequences, introns, or in intergenic regions [27]. SNPs are used as genetic signatures in populations to study the predisposition to certain traits, including diseases [29].

### The anatomy of the problem

In the era of advanced DNA sequencing tools and personal genomics, these earlier definitions of mutation and polymorphism are antiquated. Before multiple parallel sequencing was developed, it was impossible to sequence multiple times the genome of the same patient. For these reasons at that time it was required to use a reference sequence coming from the assembly of multiple genomes. In the preparation of the consensus sequence, an arbitrary threshold of 1 % was established to distinguish common (polymorphism) from rare (mutation) variants [26].

The 1 % or higher frequency associated with a polymorphism is an arbitrary number [30] recommended by scientists prior to the era of Next Gen Sequencing. The threshold being arbitrary, redefining the population itself may affect the classification, with rare variants becoming polymorphisms or polymorphisms becoming rare variants according to the population analyzed. For decades, the use of this frequency to develop population models was preferred to the use of sequencing tools, which at that time were error-prone and labor-intensive. With the advent of new sequencing technologies and the subsequent sequencing of individuals, a very different picture of population dynamics has begun to emerge. Mutations that were thought to be rare in a population have been found to exceed the frequency threshold set at 1 % [31]. Even more surprising, there is a lack of association of some of these rare mutations with human diseases. When comparing populations separated by geographic and physical barriers, a disease-causing mutation in one population is found to be harmless in another, and vice versa [32].

For instance, sickle-cell anemia is caused by a nucleotide change (SNP rs334) in a gene coding for the beta chain of the hemoglobin protein [33]. In fact, rs334 is classified as a SNP, since its minor allele frequency in the population is >1 %. The disease manifests in people who have two copies of the mutated gene (rs334(T;T) genotype). Sickle cell anemia is usually rare (<1 %) in the populations of developed nations [34]. However, the heterozygous form of the gene (rs334(A;T) genotype) is persistent in populations of Africa, India, and other developing nations, where malaria is endemic [33]. In these geographic locations, heterozygote carriers of rs334 have a survival advantage against the malaria pathogen, and therefore this beneficial mutation is passed through the offspring to succeeding generations [35–37]. Here, a rare variant, which in one population (developed nations) causes a severe disease in homozygosis, can persist in another population to confer a survival advantage as a polymorphism in heterozygosis [38]. Such exceptions are increasing and show the need to redefine the terms mutation and polymorphism. The distinction between mutation and polymorphism on the basis of their disease-causing capacity is further complicated. Although thought to be naturally occurring, recent research into SNPs has shown that they can be associated with diseases like diabetes and cancers. At least 40 SNPs have been shown to associate with type-2 diabetes alone [39]. In short, it is not possible to classify the functional role of variations according to frequency in the population or their capability to cause a disease.

### Context of personal genomics

This debate on “mutation” and “polymorphism” needs urgent evaluation in the era of Next Gen Sequencing and precision medicine. Multiple international collaborative projects like ENCODE (Encyclopedia of DNA elements) and HapMap (Haplotype Map) have ensued to map all the genes, genetic variation, and regulatory elements of the genome, to find associations with human biology, personal traits, and diseases [40].

In this climate, commercial companies like Illumina and Roche are developing advanced and robust platforms that tailor to the need of both small and large research facilities. The increasing competition among these companies has resulted in many different technologies, which are now available to facilitate new insights into genomics [11]. Similarly, advanced genomic tools and analytical software have been developed that can function independently of the particular platform. Researchers using tools like CLC genomics, Next Gene and Geno Matrix, can access and download sequencing datasets for their own streamlined research. The primary goal of such research is to look for subtle, complex, and dynamic sequence variations. The lack of consistent definitions and a uniform scientific language can hamper this upcoming field, where genomic platforms may formulate incorrect hypotheses and researchers may misinterpret data based on earlier definitions.

The problem is particularly important in the case of precision medicine and personalized treatments. For example, one of the main reasons to sequence the genome of a cancer consists in the identification of unique genetic features of cancer cells which may then be targeted with a personalized treatment [41]. Accordingly, it is required to classify the somatic mutations of the cancer cells and use such knowledge to exploit therapeutically all the differences between cancer and noncancerous cells. Therefore, in order to be treated with a targeted agent a cancer patient needs to express the target originated by the specific mutation occurring in cancer cells. However, should a difference be misclassified, it becomes possible for a polymorphism (present in all the cells of the patient) to be taken as a somatic mutation. The result could be a toxic effect, since the targeted treatment will impact both cancer and noncancerous cells carrying the same genetic variant. This problem is prevented if both germline and somatic cancer genomes would be sequenced in the same patient.

Another important reason underlying the need of such distinction is that a disease may originate with two subsequent mutations according to the two-hit hypothesis [42]. Within a population, a germline mutation (first hit) may predispose a subset of patients to a second, somatic, mutation whose effects will create the diseased phenotype [43]. In this context, in order to identify populations at risk it would be extremely helpful to distinguish between somatic and germline mutations. For example, multiple meningiomas occur in <10 % of meningioma patients. A first germline mutation in the *SMARCB1* gene will predispose to meningioma, but this will occur only when a somatic mutation in the *NF2* gene intervenes [44]. In the absence of a clear distinction between somatic and germline variants this kind of pathogenic discovery may be impossible.

This approach is now supported by a recent study. Jones et al. evaluated 815 tumor-normal paired samples coming from 15 different tumor types [45] using Next Gene Sequencing. Library preparation was performed with two methods, whole exome preparation and targeted amplification, for 111 genes. Analyses were then conducted either as if only the cancer tissue was sequenced (reference human genome assembly GRch37-lite) or taking as reference the germline DNA of the same patient. With the first analysis, the authors reported a very high rate of false-positive variants (31 % and 65 % in exome and targeted libraries, respectively). Furthermore, they identified germline mutations in 3 % of the cancers, even if they came from a cohort without family history (sporadic cancer). Now that the new sequencing technologies have dramatically reduced the cost of sequencing, precision medicine and personal genomics require that the reference of the DNA sequencing project should be obtained from the germline DNA of the same patient.

### Ongoing debate and HGVS (Human Genome Variation Society) recommendations

The ongoing debate among scientists to resolve the nomenclature mutation and polymorphism is a step in the right direction. The HGVS, an alliance of 600 members from 34 countries, incorporates discussion and recommendations to establish consensus definitions and descriptions of generic terms that are accepted worldwide. Since the early 1990s, the HGVS has been instrumental in its push to standardize the mutation nomenclature. The recommendations of the HGVS have been based on extensive discussions among scientists over the years.

The papers published on this topic for the last 20 years show that HGVS was visionary to recommend new changes and extensions based on discoveries of relatively complex variants. In 2002, several researchers tried to address this nomenclature problem and the challenges to make more inclusive definitions.A special article by Condit et al. found that mutation had become increasingly negative in connotation since its use in the biological sciences, but particularly over the course of the 20th century [22]. This negativity of the term became entrenched with radiation experiments and the use of atomic weapons during the II^nd^ world war, and later with science fiction books and movies. The paper suggested that a better term like “variation” and “alteration” might be useful, but its inconsistent usage in the scientific world makes it problematic.

More recently, additional papers have highlighted the urgency of a “consensus” guiding the selection of the sequencing methods (data collection) and reporting. These studies point out that the accurate classification of pathogenic variants requires a standardized approach and the building of data repositories including all these data [46]. In this context, Richards et al. on the behalf of the American College of Medical Genetics and Genomics (ACMG) have noted that the terms “mutation” and “polymorphism” often lead to confusion because of incorrect assumptions of pathogenic and benign effects, respectively. Thus, they recommended that both terms be replaced by the term “variant” with the following modifiers: (i) pathogenic, (ii) likely pathogenic, (iii) uncertain significance, (iv) likely benign, or (v) benign [47].

## Summary

Despite this rhetoric to better define the terms, there is no consensus in research papers or HGVS recommendations on how a mutation is different from a polymorphism. The lack of a consensus is creating a problem in the interpretation of data coming from personal genome software analysis, as described above. What is the reference? What is the threshold to distinguish common from rare DNA variants? This problem is not trivial when looking at the downstream effects. In fact, the term mutation is commonly conceived (wrongly) to carry an intrinsic negative impact on the function of a given gene.

We propose that the term “mutation” be used to indicate the result of a recent mutation event which has been detected using as a reference the germline DNA of the same individual. Therefore, a mutation would be a “DNA variant” acquired over the lifetime of an organism, i.e. a somatic mutation. In this sense, mutations are the principal causes of many diseases like cancer but are typically not inherited by their offspring. Alterations in the DNA of germ cells – sperms and eggs – can be inherited by offspring and are currently called germline mutations. In this case, the term mutation should be used only if the germline “variant” has been detected using as a reference the germline DNA of the same individual. While germline mutations can also increase the likelihood of succumbing to certain diseases, the signature of such mutations is found in each and every cell of the offspring [48]. This is because the original embryo (first cell in the body) is formed through the fusion of germ cells, from where all the somatic cells arise. So in essence, while these alterations in the parents’ germ cells are appropriately termed germline mutations, calling both somatic and germline mutations simply “mutations” seems incongruent. The variation of genotypes among individuals, inherited from parents but still present in the DNA of each cell in the body, is the classic definition of a genetic polymorphism and we propose going back to this original definition: a polymorphism occurs in a population when the observed variation from individual to individual is not maintained by recurrent mutation.

Whereas it is perhaps not unreasonable to use the term mutation for the result of a mutation event, there is no analogy that would imply using the term polymorphism for a common variant because polymorphism is a condition found in a population, not an event. Genetic polymorphism, just like any other biological polymorphism (e.g. the siphonophores) occurs when members of a species differ in form [49]. When the notion that the different forms could be genotypes rather than phenotypes was first introduced [49], the focus was on the least frequent genotype not being due to recurrent mutation, and hence the arbitrary 1 % threshold; but a genetic locus with a thousand equi-frequent alleles would be considered extremely polymorphic. Most SNPs are tri-morphic, but are appropriately called polymorphisms in contrast to being mono-morphic.

Different from diseases associated with SNPs, which are expressed in all the cells of an organism, some diseases, like cancer, are caused by genetic variations typical of a small subset of somatic cells. In order to keep the difference between the two categories of DNA variants, we propose a clear distinction between a SNP and a somatic mutation. A tumor sample and a normal tissue sample from the same individual can be sequenced for analysis of genetic variations. For statistical and computing power, additional sequences coming from buccal swabs or peripheral blood DNA can be used to sequence the germline reference of a patient (paired approach). Since the tumor samples have additional genetic changes as compared with the specific individual’s germline reference, these changes will serve as key attributes to understanding the cancer of this specific individual.

**Fig. 1:**
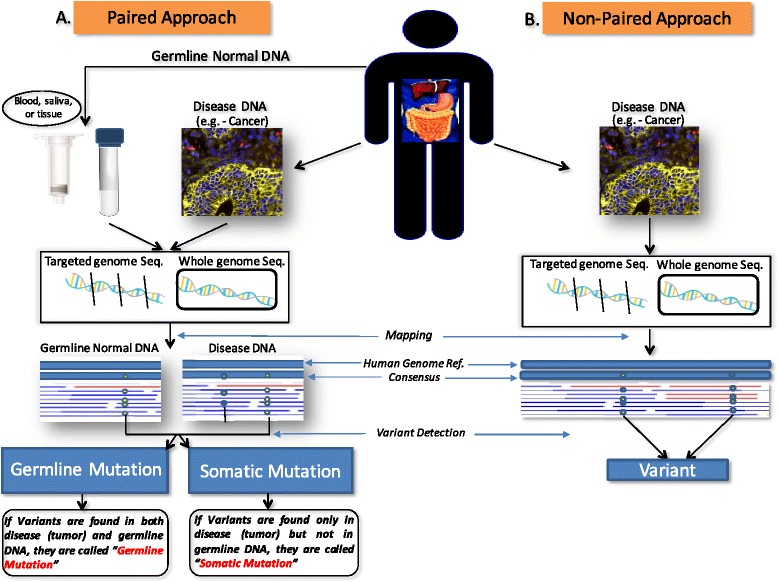
Nomenclature of variants according to sequencing design. In a paired approach (**a**), diseased (tumor) DNA and DNA from the germline (blood, saliva, or other non-diseased tissue) have been extracted and individually sequenced and mapped against a human genome reference assembly. If there are common variants found in both the tumor and germline DNA, they should be called germline mutations. If there are variants found only in tumor DNA, they should be called somatic mutations. In a non-paired approach of variant detection (**b**), only diseased DNA is extracted from the tissue of interest. The extracted DNA has been sequenced and mapped against a human genome reference assembly and differences as compared with the reference will be labeled as variants

It is possible that germline sequences between this individual and others also differ, and this would constitute a polymorphism in the population, as originally defined. The genotypes/alleles that constitute a polymorphism should be called variants but never, without attribute, simply “mutations”. In our proposal, the term “mutation” should be used only if the sequencing project used the germline reference (Fig. 1a). In this context, in order to have a mutation it is not only required to detect a variation as compared with the reference, but also the reference needs to be represented by the germline cells of the same individual. Accordingly, the term “mutation” should always be accompanied by a qualifying prefix indicating if the “mutation” occurs only in somatic cells (somatic mutation) or also in the germ line cells (germline mutation) (Fig. 1a). This would prevent mutations and polymorphisms from being incorrectly annotated in a sequencing project, with potential deleterious effects on the efficacy of genomics applied to precision medicine, as recently highlighted in recent studies [45–47].

In the case a sequencing project did not include as a reference the germ-line DNA of an individual, the term “mutation” could not be used and should be replaced by the neutral term “variant” (Fig. 1b), as previously suggested [47]. Therefore, in the sequencing report the alternative use of the term “mutation” or “variant” will also clarify which kind of reference was adopted. We anticipate that this approach will encourage the use of referencing germline DNA in a sequencing project and will allow an immediate comparison between studies that used the same referencing method. Importantly, the term “polymorphism” should only be used in the context of a population. Accordingly, this term cannot be approved to classify variants in personal genomics.

## Abbreviations

SNP: Single nucleotide polymorphism
HGVS: Human Genome Variation Society
